# Near-commensuration from Intertwined Charge Density Waves in Single-Layer TiSe_2_

**DOI:** 10.1021/acs.nanolett.5c05317

**Published:** 2026-01-06

**Authors:** Wen Wan, Maria N. Gastiasoro, Daniel Muñoz-Segovia, Paul Dreher, Miguel M. Ugeda, Fernando de Juan

**Affiliations:** † 226245 Donostia International Physics Center, P. Manuel de Lardizabal 4, 20018 Donostia-San Sebastian, Spain; ‡ Centro de Fısica de Materiales CSIC-UPV/EHU, 20018 Donostia-San Sebastián, Basque Country, Spain; § 5798 IKERBASQUE, Basque Foundation for Science, Maria Diaz de Haro 3, 48013 Bilbao, Spain

**Keywords:** Near commensurate charge density waves, 2D materials, transition metal dichalcogenides, scanning tunneling microscopy

## Abstract

When the period of an incommensurate charge density wave (ICDW) approaches a multiple of a lattice vector, the energy gain from locking the period can drive a transition into an intermediate near-commensurate (NC) state made of locally commensurate regions separated by phase slips of a complex CDW. In TiSe_2_, a paradigmatic CDW system, incommensuration is believed to be induced by carrier doping, yet its putative NC state has never been imaged. Here we observe a striking NC state in ultraclean, doped monolayers of TiSe_2_, displaying an intricate network of unidirectional domain walls. Detailed analysis reveals that these are not phase slips of a complex CDW, but rather sign-changing Ising-type domain walls of two coupled real CDWs of different symmetry. In addition, these CDWs are coupled to an unexpected nematic modulation at the lattice Bragg peaks. TiSe_2_ is thus a rare example of a NC-CDW of two intertwined real modulations with nematicity.

## Graphical abstract

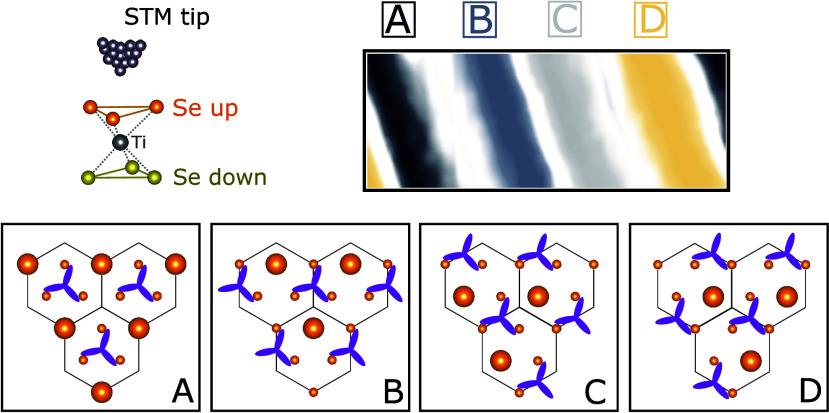

Understanding how incommensurate charge density wave (ICDW) states lock to the crystal lattice in an incommensurate to commensurate (I–C) transition is a fascinating problem with profound implications on how other ordered states such as superconductivity emerge in their presence.^1^ Due to the energetics of lattice locking, the I–C transition typically proceeds via an intermediate near-commensurate (NC) phase characterized by locally commensurate domains separated by domain walls (see phase diagram in Figure 1b). The nature of these domain walls and of the I–C transition itself depends on whether the CDW order parameter is real or complex, which is determined by the commensurate CDW (CCDW) period.^2^ In a one-dimensional (1D) CCDW with wavevector $Q=\frac{2\pi }{Na}$ and lattice constant *a*, lattice translations for *N* > 2 are represented by a phase factor e^*i*2π/*N*^ and the order parameter is complex. In this case, the NC state has approximately constant amplitude but shows regions of constant phase separated by phase slips known as discommensurations,^3^ akin to domain walls of easy-plane ferromagnets. If the CCDW has period *N* = 2 however, translations are represented by a sign change (e^*i*2π/*N*^ = −1) and the order parameter is real. In this case, only real sign-changing domain walls where the order parameter goes through zero are possible, akin to domain walls in Ising ferromagnets (see SI).

**Figure 1:**
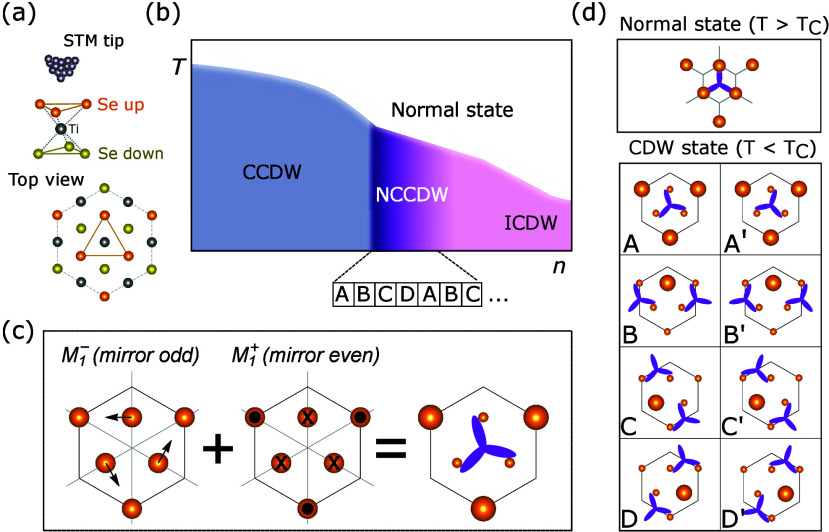
CCDW and NCCDW in monolayer TiSe_2_. (a) Side- and top-view of the crystal structure. (b) Schematic phase diagram of the doping (n)-induced NCCDW transition. (c) Sketch of the displacements of the upper Se atoms corresponding to primary (*M*_1_^–^, mirror odd) and secondary (*M*_1_^+^, mirror even) order parameters of the transition (mirror planes shown as thin gray lines). The sketch on the right shows the resulting motif commonly observed in STM, with one brighter Se atom and three dimmer ones, which make a slightly rotated propeller-like feature (purple). (d) The eight possible ground states of the 2 × 2 CCDW. The structure of the NC state in panel b is shown below the phase diagram as a 1D train of ABCD domains.

TiSe_2_ (Figure 1a) is a paradigmatic example of one such *N* = 2 triple-*Q* CDW, which can be driven through a C–I transition by electronic doping. The bulk CDW wavevector is $\mathrm{Q⃗}=2\pi \left(\frac{1}{2a},0,\frac{1}{2c}\right)$ and Cu doping^4^ or applying pressure^5^ leads to an out-of-plane incommensurate CDW state which becomes superconducting at *T*_C_ ∼ 2–4 K.^6^ In-plane incommensuration is not resolved in X-rays, but STM studies in Cu- and Pt-doped samples do observe in-plane CDW domain walls^7–11^ with a disordered spatial distribution likely dominated by CDW pinning by the Cu dopants, and indirect transport signatures of incommensuration were reported in gated thin films.^12^ Direct evidence of an NC state, i.e., a long-range ordered array of in-plane domain walls, remains lacking as this would require imaging a homogeneously doped sample without disorder. To disentangle the more complex out-of-plane incommensuration, this should ideally be done in the single-layer limit, where the *N* = 2 CDW remains essentially unchanged from the bulk.^13–19^

In this Letter, we demonstrate that a doped monolayer of TiSe_2_ exhibits an intrinsic in-plane NCCDW state with a wavelength of 20 nm, which remains coherent over hundreds of nanometers. We uncover the spatial structure of the NC state via high-resolution STM measurements combined with standard phase-locking techniques and show that it is made of a train of intertwined Ising domain walls of two independent order parameters with opposite mirror parity, shown schematically in Figure 1c. These domain walls concatenate four CDW domains out of the eight available ones (A through D′ in Figure 1d) in a periodic arrangement. Our analysis also reveals the existence of a strong nematic modulation of the lattice coupled to the domain wall train. This complex NC state is naturally accounted for by Ginzburg–Landau theory, including the order and number of the domains and their coupling. Our work clearly shows how TiSe_2_ proceeds through the doping-driven C–I phase transition via a NC state of Ising domains.

## Extracting CDW Order Parameters

Our experiments were carried out on a nearly full monolayer of 1T-TiSe_2_ grown on epitaxial bilayer graphene (BLG), as sketched in Figure 2a. Our samples display remarkable uniformity with single-crystal domains several hundreds of nanometers in size (Figure 2b) and a density of point defects below 1 × 10^12^ cm^–2^. A typical STM d*I*/d*V* spectrum, showing the low-lying electronic structure of single-layer TiSe_2_, is shown in Figure 2c (for a larger energy range, see SI). Three step-like features for occupied states are observed at *V*_s_ = −215 ± 5 mV (labeled *V*_1_), *V*_s_ = −365 ± 5 mV (labeled *V*_2_), and a subtler step with opposite orientation (see inset) labeled *C*_1_ is observed at *V*_s_ = −65 ± 5 mV. These features match the band structure of monolayer TiSe_2_ in the CDW phase,^13^ which shows an electron band of Ti-3d character (back-folded from *M⃗*_*n*_) and two spin–orbit split Se-4p hole bands (*E*_SOC_ = 150 ± 10 meV) (Figure 2d) separated by the CDW gap (*E*_G_ = 150 ± 10 meV).^20–25^ This identification is also supported by previous angle-resolved photoemission spectroscopy (ARPES) measurements,^13^ which revealed a small electron doping because the d-electron band is partially filled. This likely originates from charge transfer from the substrate, as demonstrated recently by comparing TiSe_2_ monolayers grown on different substrates.^26^

**Figure 2:**
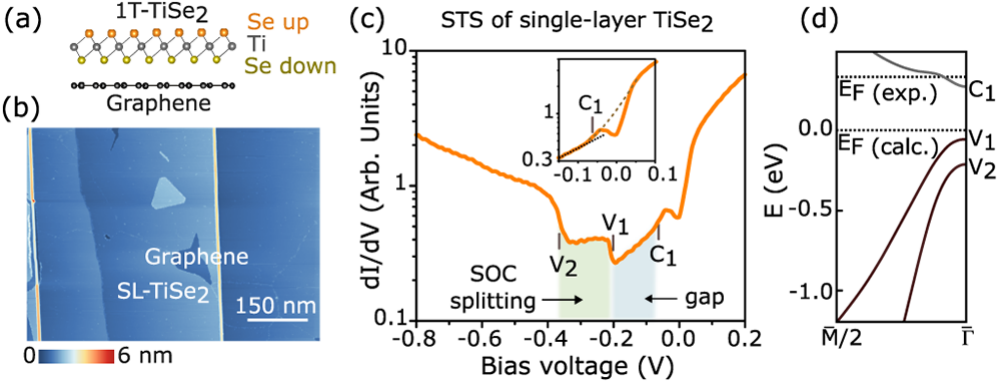
(a) Side-view sketch of monolayer TiSe_2_ on graphene. (b) Large-scale STM image of nearly one layer of TiSe_2_ on BLG/SiC(0001) (*V*_s_ = 2.4 V, *I*_t_ = 15 pA). (c) Typical STM d*I*/d*V* spectrum acquired on monolayer TiSe_2_/BLG showing electronic features *V*_1_, *V*_2_, and *C*_1_ (*I*_t_ = 2 nA). The colored shadows below the curve indicate the SOC splitting (green) and the gap (blue). Inset shows a zoom around the *C*_1_ feature. (d) Calculated band structure of the isolated monolayer TiSe_2_ in the CDW 2 × 2 phase (adapted from Chen et al., ref 13, licensed under CC BY 4.0). The position of the experimental *E*_F_ is also indicated.

Our atomically resolved STM images of the CDW state show the expected 2 × 2 order at small scales (Figure 3a), where the CDW unit cell (white hexagon) is doubled in both lattice directions compared to the crystal unit cell (green hexagon). The corresponding Fast Fourier transform (FFT) in Figure 3b shows clear superlattice Bragg peaks at both *Q⃗*_*n*_= *M⃗*_*n*_ = *G⃗*_*n*_/2 (*n* = 1, 2, 3) and the higher harmonic *M⃗*_*n*_^′^ = *M⃗*_*n*_ + *G⃗*_*n*–1_ (yellow and orange circles, respectively), which are the signature of 2 × 2 order. While this modulation seen in STM has traditionally been identified^7–11,27–30^ as resulting from the 2 × 2 lattice distortion observed in neutron scattering,^31^ we now show that the STM modulation actually reflects the existence of two CDW order parameters of different symmetry.

**Figure 3:**
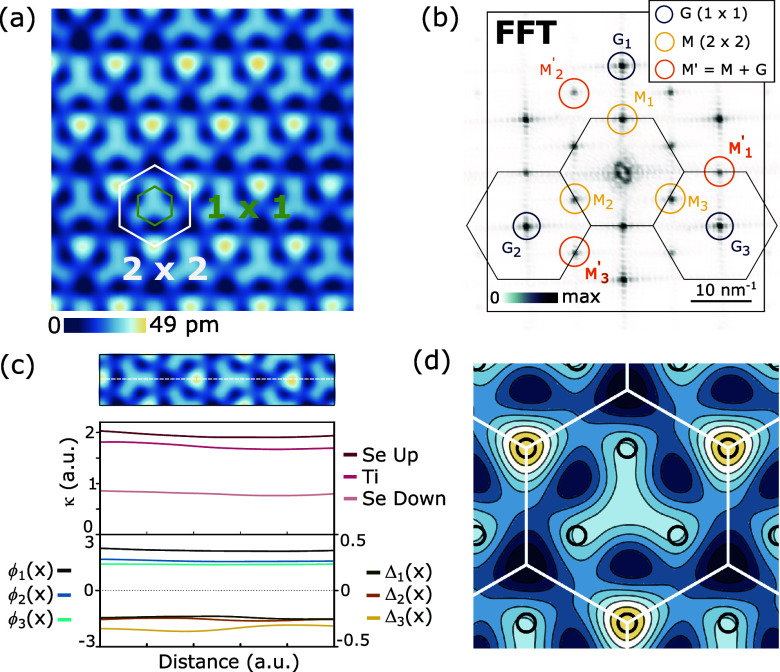
(a) Atomically resolved STM image of monolayer TiSe_2_ in the CDW state showing the 2 × 2 reconstruction (*V*_s_ = 0.1 V, *I*_t_ = 1.5 nA). The atomic and CDW unit cells are indicated in green and white, respectively. (b) FFT of the STM image in panel a, showing CDW Bragg peaks at *M⃗*_*n*_ and *M⃗*_*n*_^′^. (c) Modulus $\kappa =\sqrt{\frac{1}{3}\underset{n}{\sum }{\left(\phi_{n}^{\alpha }\right)}^{2}}$ for α = Se-Up, Ti, Se-Down (upper plot) and extracted order parameters Δ_*n*_(*x*) and ϕ_*n*_(*x*) ≡ ϕ_*n*_^SeUp^(*x*) from the CDW peak (lower plot) plotted along the dashed white line in the zoomed-in region shown above. (d) Real-space contour plot of ρ­(*x⃗*) reconstructed by taking averaged values of the order parameters in panel c showing the slightly counterclockwise rotated propeller when Δ_1_Δ_2_Δ_3_ < 0.

The primary CDW order parameter of monolayer TiSe_2_ (point group symmetry *D*_3*d*_) is a transverse phonon of symmetry *M*_1_^–^ ^31^ (irrep *A*_*u*_ of the little group *C*_2*h*_ at *M*), which is odd under both inversion and the mirror plane σ_*v*_ parallel to the corresponding *M⃗*_*n*_ vector. A depiction of the triple-*Q* *M*_1_^–^ phonon distortion is shown in Figure 1c, with in-plane black arrows representing the displacements of the top Se layer. These displacements give rise to a rotated propeller-like motif commonly observed in STM experiments, represented in purple in the figure. A secondary CDW order parameter of *M*_1_^+^ symmetry, representing a modulation where one out of four Se atoms rises upward, is symmetry allowed and condenses simultaneously at the CDW transition.^32^ The corresponding *M*_1_^+^ distortion, even under both inversion and mirror σ_*v*_, is also depicted in Figure 1c (out-of-plane black arrows). Note that in the monolayer, an *M*_1_^–^ distortion generically makes the ground state chiral,^33^ unlike in the bulk where *L*_1_^–^ always preserves an inversion center between layers.

In our monolayer samples, the *M*_1_^–^ and *M*_1_^+^ modulations can be distinguished in STM by their mirror parity as follows. The charge density in the CDW state is approximately given by ρ­(*x*) = 2Re­[∑_*n*_ *A*_*n*_^*G*^ e^*iG⃗*_*n*_ *x⃗*^ + *A*_*n*_^*M*^ e^*iM⃗*_*n*_ *x⃗*^ + *A*_*n*_^*M* ^′^^ e^*iM⃗*_*n*_^′^ *x⃗*^], where *A*_*n*_^*q*^ are complex numbers at the *q⃗* = *G⃗*_*n*_, *M⃗*_*n*_, and *M⃗*_*n*_^′^ FFT peaks. Because lattice translations act as real numbers e^*iq⃗*_*i*_ *a⃗*^ = ±1 for any *q⃗*_*i*_, the real and imaginary parts of *A*_*n*_^*q*^ encode *independent* 2 × 2 modulations, which may even have different symmetry. Since *M⃗*_*n*_ (*M⃗*_*n*_^′^) is parallel (perpendicular) to σ_*v*_, the modulation e^*iM⃗*_*n*_ *x⃗*^ is invariant under σ_*v*_, while for *M⃗*_*n*_^′^, e^*iM⃗*_*n*_^′^ *x⃗*^ → e^–*iM⃗*_*n*_^′^ *x⃗*^. This implies that Re*A*_*n*_^*M*^, Im*A*_*n*_^*M*^ and Re*A*_*n*_^*M* ^′^^ all represent mirror *even* 2 × 2 modulations, while only Im*A*_*n*_^*M* ^′^^ represents a mirror *odd* 2 × 2 modulation. Hence, the primary CDW order parameter, which we call Δ_*n*_, can only be Δ_*n*_ = Im*A*_*n*_^*M* ^′^^, which is sensitive to the small in-plane modulations of the Se atoms of symmetry *M*_1_^–^. The other three mirror even signals (Re*A*_*n*_^*M*^, Im*A*_*n*_^*M*^, Re*A*_*n*_^*M* ^′^^) can be rearranged into three *M*_1_^+^ order parameters ϕ_*n*_^α^ showing the 1-in-4 pattern localized at the upper Se (ϕ_*n*_^SeUp^), Ti (ϕ_*n*_^Ti^), and lower Se (ϕ_*n*_^SeDo^) sites, respectively (Figure 1a; See SI).

To extract all these order parameters, we implemented the Lawler–Fujita^34^ (LF) algorithm to produce corrected images in a perfect registry with the lattice, which allows extraction of the complex numbers *A*_*n*_^*M*^ and *A*_*n*_^*M* ^′^^ with well-defined phases (see SI). Figure 3c shows a 1D cut of the extracted *M*_1_^+^ secondary order parameters ϕ_*n*_^SeUp^, ϕ_*n*_^Ti^, and ϕ_*n*_^SeDo^, showing the dominance of ϕ_*n*_^SeUp^ as this layer is the closest to the STM tip. Because of this, from now on, we only consider ϕ_*n*_ ≡ ϕ_*n*_^SeUp^ as the secondary CDW order parameter. The extracted primary and secondary order parameters, Δ_*n*_ and ϕ_*n*_, along the same 1D cut are also shown in Figure 3c, revealing finite *n* = 1, 2, 3 components of the triple-*Q* CDW state.

As a check, in Figure 3d we reconstruct an approximate ρ­(*x*) from the spatially averaged order parameters obtained from Figure 3c (see SI). In remarkable agreement with the original image (Figure 3a), Figure 3d shows the same weakly rotated propeller-like shape and the 1-in-4 bright feature of the Se atoms. The rotation of this propeller reflects the existence of Δ_*n*_, with its chirality (clockwise vs counterclockwise) given by the sign of Δ_1_Δ_2_Δ_3_, while the 1-in-4 bright upper Se atom originates from ϕ_*n*_. The four choices in the CDW unit cell for the propeller center, determined by the signs of Δ_*n*_ and ϕ_*n*_, make eight possible CCDW ground states, which we label A, B, C, D for one chirality and A′, B′, C′, D′ for the other (represented in Figure 1d) and Figure 3c corresponds to state A. A faithful extraction of the primary Δ_*n*_ and secondary ϕ_*n*_ order parameters, performed for the first time here, is crucial to understand the symmetry and domain structure of the CDW, including in incommensurate states.

## Near-commensurate CDW State

Our main experimental result is now revealed by our high-resolution topography images taken over very large scales, which show a highly inhomogeneous 2 × 2 CDW displaying a striking long-range ordering of domains characteristic of a near-commensurate phase. Figure 4a shows an atomically resolved STM image of 153 nm × 153 nm in size, where long striped regions dominate the topography, forming an intricate pattern. The FFT of this image (Figure 4b) reveals characteristic CDW peaks at *M⃗*_*n*_ and *M⃗*_*n*_^′^ as observed for a single domain (Figure 3b). While correcting the phase drift with the LF algorithm in such a large image is not possible, the Fourier-filtered amplitude (insensitive to phase drift) of the *M⃗*_*n*_ peaks (Figure 4c), reveals that the long-wavelength topography modulation indeed comes from a modulation of the CDW and shows that the wavelength appears halved compared to the topography. We systematically observed these patterns in tens of different regions in several TiSe_2_/BLG samples (see SI), enabling us to extract an averaged periodicity of λ = 20 ± 2 nm for the modulation induced perpendicular to the domain walls. Remarkably, the incommensuration is single-*q* in character (i.e., the modulation produces 1D stripes and is not a 2D network as previously believed).^12,35^ The 1D trains of domains in Figure 4c form “superdomains” that can be distinguished by the modulation vector *q⃗*_NC_, roughly parallel to one of the CDW wavevectors *Q⃗*_*n*_. A zoom of the three types of superdomains (Figure 4d) reveals a finer feature of domain walls: two classes of them, wide and narrow, alternate within the superdomain train.

**Figure 4:**
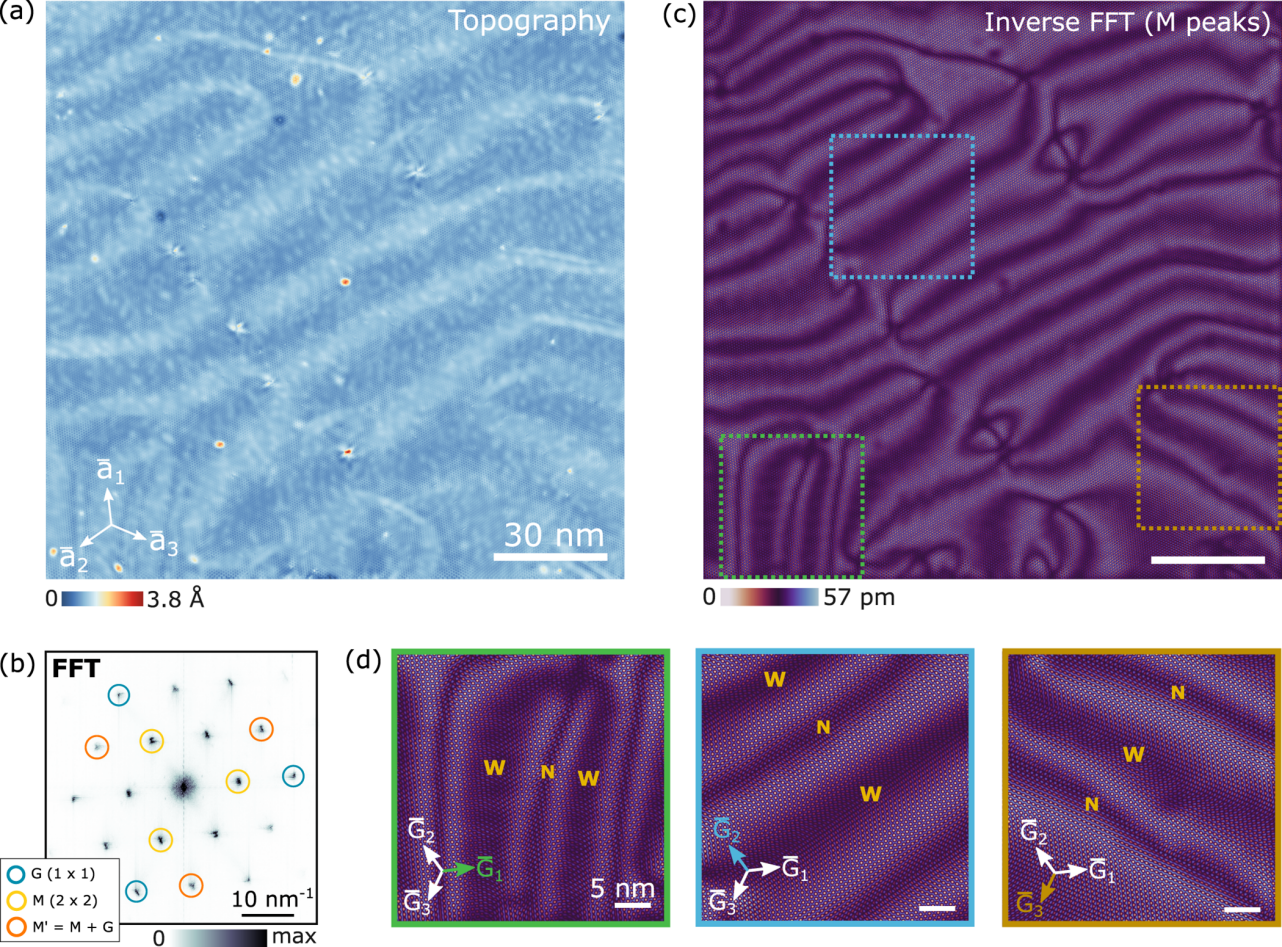
Near-commensurate CDW state. (a) Atomically resolved STM topography of a 153 nm × 153 nm region (*V*_s_ = −0.05 V, *I*_t_ = 60 pA). Lattice vectors are shown as white arrows. (b) FFT image of panel a. (c) Inverse FFT of the amplitude of the CDW *M⃗*_*n*_ peaks from panel b (see SI for *M⃗*_*n*_^′^). A network of 1D trains of domains (high amplitude) separated by domain walls (low amplitude) with an average spacing of 20 nm is observed. (d) Zoom-in of three regions shown in colored squares in panel c, showing that domain walls are approximately perpendicular to *G⃗*_*n*_ and alternate between wide (W) and narrow (N) types.

The behavior of the CDW in the single-layer limit sharply contrasts both with pristine bulk TiSe_2_, where the CDW shows a commensurate, single-domain phase,^28,29^ and with bulk doped samples, which show short-range, disordered domain walls.^7–11^ The ordered domain structure in the single layer cannot be attributed to disorder given the low density of defects of our films (see SI) and instead represents an intrinsic near-commensurate state. These patterns can still be seen at 77 K but are not present above *T*_CDW_ ≈ 230 K, as seen in our STM images at room temperature (see SI), which also ensures that they are not extrinsic and originate from the CDW incommensuration.

## Intertwined CDW order parameters

To analyze the order parameter structure of the NC state, we selected an STM image with a single superdomain (Figure 5a) where *q⃗*_NC_ ∝ *G⃗*_2_. The extracted order parameters are shown in Figure 5b, which reveals another striking feature: the modulation of the three Δ_*n*_ components is different, approximately following Δ_2_ ∝ sin­(2*q⃗*_NC_ *x⃗*), Δ_1_ ∝ sin­(*q⃗*_NC_ *q⃗*) + *c* sin­(3*q⃗*_NC_ *x⃗*), and Δ_3_ ∝ cos­(*q⃗*_NC_ *x⃗*) – *c* cos­(3*q⃗*_NC_ *x⃗*) with |*c*| < 1. The secondary order parameter components follow a similar pattern with the opposite sign, ϕ_*n*_ = −Δ_*n*_. This leads to sign alternation of domains which produces the pattern “ABCD” illustrated in Figure 5d, concatenating the four CCDW states with counterclockwise chirality Δ_1_Δ_2_Δ_3_ < 0 (left column in Figure 1d). A zoom around a domain wall in Figure 5c shows how a given pattern of sign changes shifts the center of the CDW by one atomic unit cell: in this case, the transition from state B to C corresponds to a translation by lattice vector *a⃗*_3_.

**Figure 5:**
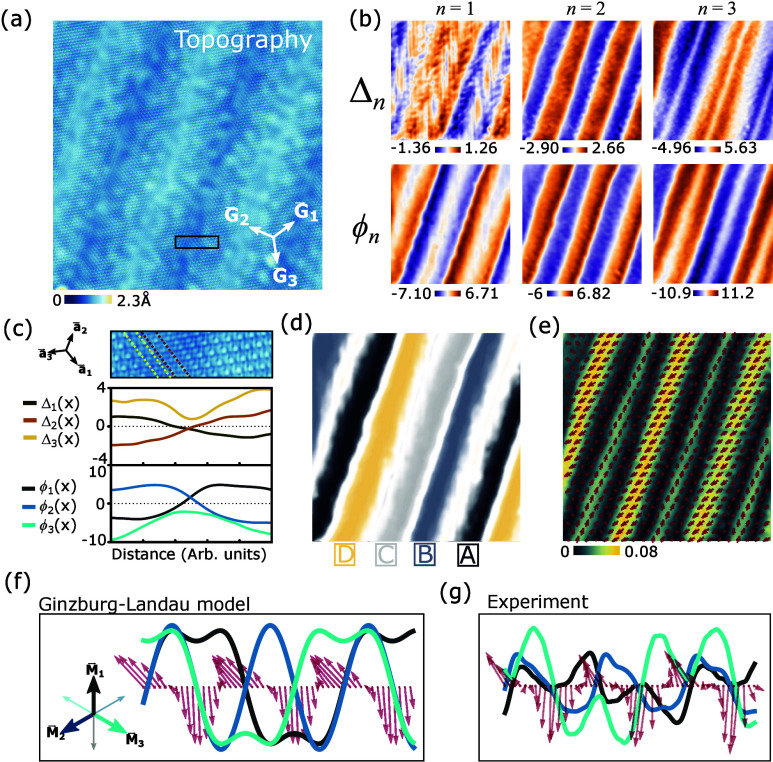
Ising domains of intertwined CDW order parameters. (a) High-resolution topograph of a superdomain with *q⃗*_NC_ ∝ *G⃗*_2_ after LF correction (*V*_s_ = −0.05 V, *I*_t_ = 40 pA). (b) Extracted order parameters, ϕ_*n*_(*x⃗*) and Δ_*n*_(*x⃗*). Note that Δ_2_ oscillates twice as fast as Δ_1,3_ and ϕ_*n*_ ∼ −Δ_*n*_. (c) Close-up of a domain wall in the STM image in panel a (black box) and corresponding 1D cuts of the order parameters. The domain wall connects state C (left) to B (right), according to the convention in Figure 1d. This corresponds to a real space shift by *a⃗*_3_ (illustrated by dashed lines). (d) Color map of domains extracted from panel b, which shows the train ABCD. (e) Nematicity map *N⃗*(*x⃗*) obtained from the lattice Bragg peaks (color maps |*N⃗*(*x⃗*)| and the arrows indicate its direction). *N⃗*(*x⃗*) lives on domain walls and alternates its direction between *G⃗*_1_ (wide domain walls) and *G⃗*_3_ (narrow domain walls). (f, g) A 1D cut of ϕ_*n*_(*q⃗*_NC_ *x⃗*) (full lines) and *N⃗*(*q⃗*_NC_ *x⃗*) (red arrows) (f) from the GL theory and (g) from the STM experiment.

The LF analysis also reveals a very strong modulation of the imaginary lattice Bragg peaks Im*A*_*n*_^*G*^, while Re*A*_*n*_^*G*^ remains smoother. To track this modulation, we define a nematic order parameter

(Nx,Ny)=Im[−32(A2G−A3G),A1G−A2G/2−A3G/2]

that vanishes above *T*_CDW_ and selects a preferred axis as the CDW sets in. Figure 5e shows its intensity and direction dependence across the single superdomain. Intriguingly, this nematicity is strongest in the domain walls, where the CDW is weakest. Furthermore, it points along *G⃗*_1_ for wide domain walls and along *G⃗*_3_ for narrow ones, showing it is strongly coupled to the CDW modulation.

The observed domain wall phenomenology can be understood from the Ginzburg–Landau (GL) theory of coupled CDW order parameters Δ_*n*_ and ϕ_*n*_, whose energy density is $F=F_{\mathrm{CCDW}}+F_{\partial }^{\Delta }+F_{\partial }^{\phi }+F_{\partial }^{\Delta \phi }+F_{N}$ . The first term governs the CCDW phase

$$F_{\mathrm{CCDW}}=\underset{n}{\sum }F_{\Delta }+F_{\phi }+b_{\phi }\phi_{1}\phi_{2}\phi_{3}+b_{\Delta \phi }\phi_{n}\Delta_{n+1}\Delta_{n+2}$$

where $F_{\vartheta }=a_{\vartheta }{\vartheta_{n}}^{2}+c_{\vartheta }{\vartheta_{n}}^{4}$ for order parameters ϑ = Δ,ϕ, and we have only included a single quartic term for simplicity. Finite primary Δ_*n*_ develop when *a*_Δ_ < 0, which leads to finite secondary ϕ_*n*_ through a finite *b*_Δϕ_. Because ϕ_*n*_ is mirror even, a cubic term *b*_ϕ_ is allowed; this coefficient selects the sign of ϕ_1_ϕ_2_ϕ_3_. Indeed, we have experimentally observed only domains where ϕ_1_ϕ_2_ϕ_3_ > 0 that fixes *b*_ϕ_ < 0, whereas both signs of Δ_1_Δ_2_Δ_3_ are observed, in agreement with the absence of a cubic term for Δ_*n*_. The derivative terms which drive the incommensuration take the form $F_{\partial }^{\vartheta }=\underset{n,i}{\sum }d_{1}^{\vartheta }{\left(\partial_{i}\vartheta_{n}\right)}^{2}+d_{3}^{\vartheta }{\left(\left({\partial_{x}}^{2}+{\partial_{y}}^{2}\right)\vartheta_{n}\right)}^{2}$ for ϑ = Δ, ϕ, and $F_{\partial }^{\Delta \phi }=\underset{n}{\sum }d_{1}^{\Delta \phi }{\mathrm{Q⃗}}_{n}\times \left(\Delta_{n}\mathrm{\partial ⃗}\phi_{n}-\phi_{n}\mathrm{\partial ⃗}\Delta_{n}\right)$ . Interestingly, the fact that Δ_*n*_ and ϕ_*n*_ are locked together, Δ_*n*_ ∝ ±ϕ_*n*_, suggests that the incommensuration is not driven by a linear derivative term *d*_1_^Δϕ^ (as in McMillan theory^3^) but instead by *d*_1_^Δ^ > 0, *d*_3_^Δ^ > 0, since *d*_1_^Δ^(∂_*i*_Δ_*n*_)^2^ = −*d*_1_^Δ^(*q*_*i*_Δ_*n*_)^2^ produces an energy gain for finite *q*_*i*_. This is characteristic of Ising domain walls in incommensurate systems with real order parameters. Finally, the coupling to the nematic order parameter is

$$F_{N}=a_{N}(-\frac{\sqrt{3}}{2}\left(\Delta_{2}^{2}-\Delta_{3}^{2}\right),\Delta_{1}^{2}-\frac{\Delta_{2}}{2}-\frac{\Delta_{3}}{2})\cdot \left(N_{x},N_{y}\right)$$

By minimizing this free energy following ref 36, we found a solution, shown in Figure 5f, which qualitatively reproduces all experimental observations, shown in the same format in Figure 5g. First, the harmonic content where one CDW order parameter component modulates with 2*q⃗*_NC_ while the other two modulate with *q⃗*_NC_ and 3*q⃗*_NC_ is exactly reproduced and is generated by cubic couplings *b*_Δϕ_ and *b*_ϕ_. This reveals the important role of ϕ_*n*_ in the NC state, since Δ_*n*_ does not have a cubic term and hence this solution is impossible for Δ_*n*_ alone. Second, the nematic order parameter modulation *N⃗*(*q⃗*_NC_ *x⃗*) (red arrows in Figure 5f,g) is also reproduced. Inside domains, the local Δ_*n*_ configuration is approximately threefold symmetric, and the coupling to nematicity vanishes. However, at domain walls, one of the three Δ_*n*_ components dominates, and the coupling in $F_{N}$ is maximal when *N⃗* points along the corresponding *G⃗*_*n*_. This produces the alternation of the nematic direction in consecutive domain walls. Third, the existence of wide and narrow domain walls in Figure 4d can be explained by noting that *q⃗*_NC_ is not exactly parallel to a *G⃗*_*n*_ vector by a very small angle and that as extra quadratic derivative coupling is symmetry allowed

$$F_{\partial }^{\mathrm{anis}}=d_{2}^{\Delta }{\mathrm{Q⃗}}_{n}\cdot \left(2\left(\partial_{x}\Delta_{n}\right)\left(\partial_{y}\Delta_{n}\right),{\left(\partial_{x}\Delta_{n}\right)}^{2}-{\left(\partial_{y}\Delta_{n}\right)}^{2}\right)$$

which reflects the threefold anisotropy of the lattice. In a superdomain with *q⃗*_NC_ ∝ *G⃗*_2_, domain walls alternate their nematicity between *G⃗*_1_ and *G⃗*_3_ (i.e., Δ_1_ or Δ_3_ dominates at the domain wall). If *q⃗*_NC_ is not exactly parallel to *G⃗*_2_, *d*_2_^Δ^ gives different energies to domain walls with nematicity pointing along *G⃗*_1_ vs *G⃗*_3_, naturally making the most favored one wider and the other narrower.

## Discussion

By combining rigorous symmetry analysis with phase locking techniques, our work has established the long-sought structure of the near-commensurate state in monolayer TiSe_2_. The NC state is unidirectional and formed by the modulation of two intertwined real CDW order parameters with Ising domain walls, in accordance with its parent 2 × 2 CDW state. The free energy analysis shows the coupling to the secondary order parameter to be crucial to obtain the observed NC states, as the established primary order parameter of TiSe_2_ would not support such states on its own. Finally, the NC state displays strong nematic coupling at domain walls.

While we have described the local domains as approximately threefold symmetric, the fact that NC superdomains are unidirectional implies that a small amount of anisotropy is naturally expected also within local domains. Because of this, our data on the NC state does not determine whether the parent commensurate state is threefold symmetric.^37,38^ Nematicity only occurs within domain walls and is probably not expected in the undoped commensurate state. Similarly, while in our monolayer TiSe_2_ samples inversion symmetry is broken by the *M*_1_^–^ CDW, this has no implications on whether bulk TiSe_2_ with a primary *L*_1_^–^ CDW preserves this symmetry.^39–42^ The precise knowledge of the NC state also sets strong constraints for the interplay of CDW and superconductivity in TiSe_2_, as for example the Little–Parks effect^12^ should be very different for 2D vs 1D domain wall networks. Finally, by revealing the nature of the NC state, our work more generally provides a key piece to understand the global phase diagram of period doubling CDWs, including their I–C transitions, emphasizing the importance of Ising domain walls and the difference with the usual NC states observed in complex CDWs with *N* > 2.

## Supplementary Material



## Notes

The SI contains a comparison of real vs complex CDW domain walls, extended sample characterization and large scale electronic structure, more details on the structure of primary and secondary order parameters and phase analysis, additional characterization of the NC state and its temperature and magnetic field dependence. SI contains refs 43 and 44 . (PDF)
