# Supplementary material for: Near-commensuration from Intertwined Charge Density Waves in Single-Layer TiSe2
Source: Nano Lett. 2026 Jan 6;26(2):816–22. doi: 10.1021/acs.nanolett.5c05317 (PMC12833846; doi:10.1021/acs.nanolett.5c05317)
Supplement: Supplementary file 1 [file nl5c05317_si_001.pdf]

Supplementary Materials for

Near-commensuration from intertwined charge density waves in single-layer $TiSe_2$

Wen Wan[1], Maria N. Gastiasoro*[1], Daniel Muñoz-Segovia[1], Paul Dreher[1], Miguel M. Ugeda*[,1,2,3] and Fernando de Juan*[,1,3]

[1]*Donostia International Physics Center, Paseo Manuel de Lardizábal 4, 20018 Donostia-San Sebastián, Spain*

[2]*Centro de Física de Materiales, Paseo Manuel de Lardizábal 5, 20018 San Sebastián, Spain.*

[3]*Ikerbasque, Basque Foundation for Science, Plaza Euskadi 5, 48009 Bilbao, Spain*

**Corresponding authors: maria.ngastiasoro@dipc.org, mmugeda@dipc.org, fernando.dejuan@dipc.org*

This PDF file includes:

1. Complex vs real CDW order parameters

2. Sample preparation and initial characterization of single-layer $TiSe_2$

3. Large-scale electronic structure of single-layer $TiSe_2$

4. Primary and secondary order parameters of the CDW

5. STM imaging of the CDW pattern

6. Magnetic-field insensitivity of the CDW pattern

7. Temperature dependence of the CDW patterns

8. Fourier-filtered STM imaging of relevant periodicities

1. Complex vs real CDW order parameters

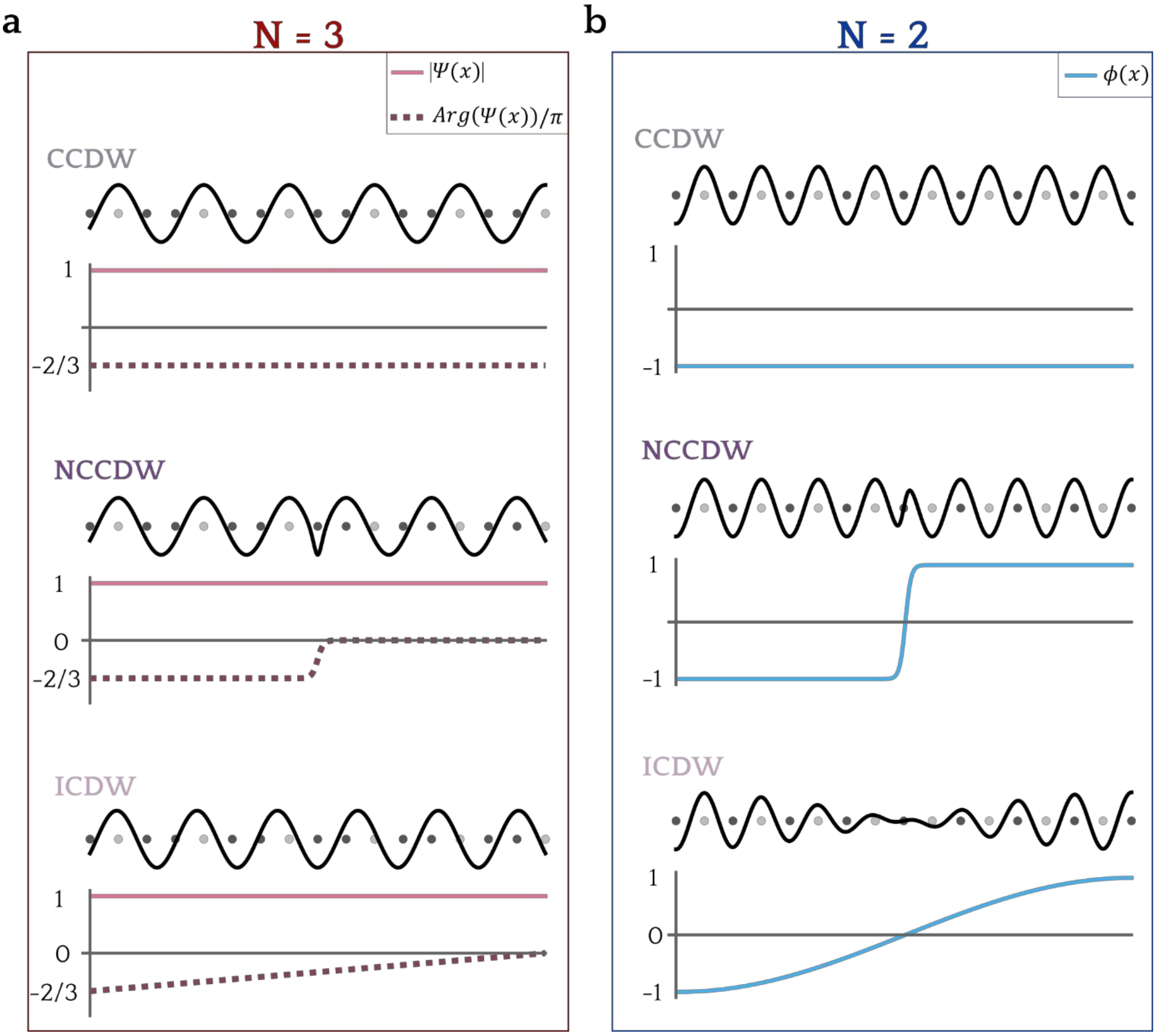

Fig. S1 | Complex vs. real order parameters in C-NC-I CDW transitions with $Q = 2\pi/Na$. a, Example of a 1D CDW with $Q = 2\pi/3a$ ($N = 3$), where the order parameter is complex and lattice translations are represented by a phase, $\Psi(x+a) = e^{iQa}\Psi(x) = e^{i2\pi/3}\Psi$. The top panel shows the commensurate state. An incommensurate state can be represented as a state where the phase grows linearly with constant amplitude (bottom panel). This state is unstable to a near-commensurate CDW (middle panel) where the phase grows via sharper phase slips known as discommensurations. **b**, A CDW with $Q = \pi/a$ ($N = 2$), where the real and imaginary parts of the Fourier transformed charge density represent independent real order parameters with different symmetry. The top panel shows the CCDW for the real part ϕ. In this case lattice translations are represented by a sign change $\phi(x+a) = e^{iQa}\phi(x) = -\phi(x)$. An incommensurate state (bottom panel) is described by a smoothly modulating ϕ, and this state is unstable to an NC state where ϕ is locally constant within domains and has sharper sign changes between them known as Ising domain walls.

2. Sample preparation and initial characterization of single-layer $TiSe_2$

Single-layer $TiSe_2$ samples were epitaxially grown on bilayer graphene (BLG) on SiC(0001) substrates. First, uniform bilayer graphene was prepared by direct annealing 6H-SiC (0001) at a temperature around 1400 °C for 35 min. For the growth of monolayer $TiSe_2$, we co-evaporated high-purity Ti (99.95%) and Se (99.999%) in our home-made molecular beam epitaxy (MBE) system under base pressure of ~5 $\times 10^{-10}$ mbar. The flux ratio between Ti and Se is 1:30. During the growth, the temperature of BLG/SiC(0001) substrates were maintained at 450℃ and the growth rate was ~20 minutes/monolayer. After the growth of monolayer $TiSe_2$, the samples were kept annealed in the Se environment for 2 minutes to minimize the presence of atomic vacancies, and then immediately cooled down to room temperature. In-situ RHEED was used for monitoring the growth process. Atomic Force Microscopy at ambient conditions was routinely used to optimize the morphology, domain sizes, coverage and cleanliness of the $TiSe_2$ islands (see Supplementary Fig. 2). The samples used for AFM characterization were not further used for STM measurements. For further transfer of the samples from our MBE to the UHV-STM chamber, a Se capping layer with a thickness of ~10 nm was deposited on the surface to protect the film from contamination during transport. The Se capping layer was subsequently removed in the UHV-STM by annealing the sample at 300℃ for 40 minutes prior to the STM measurements.

STM/STS measurements were performed on a commercial STM (USM1300, Unisoku) operated at 4.2 K using Pt/Ir tips, which were calibrated on Au(111) prior to the STM/STS measurements. Standard lock-in technique was employed for STS data acquisition, using AC modulation voltages $V_{a.c.}$ ~1 mV at f = 833 Hz. All STM/STS data were post-processed and analyzed employing the freeware WSxM [S1].

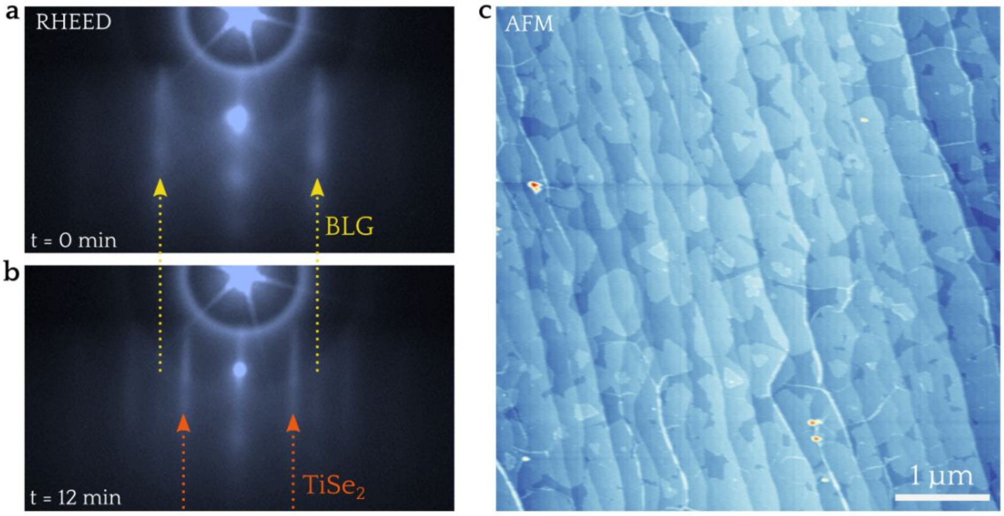

Fig. S2 | Initial characterization of the $TiSe_2$ monolayers. a, RHEED pattern after the growth of BLG/SiC(0001). Yellow lines indicate the diffraction features of BLG. The growth of the $TiSe_2$ monolayers gradually leads to new diffraction lines (red arrows) along with the attenuation of the BLG pattern, as shown in **b**. **c**, Amplitude-modulation AFM image showing the large-scale morphology of our single-layer $TiSe_2$ films on BLG/SiC(0001).

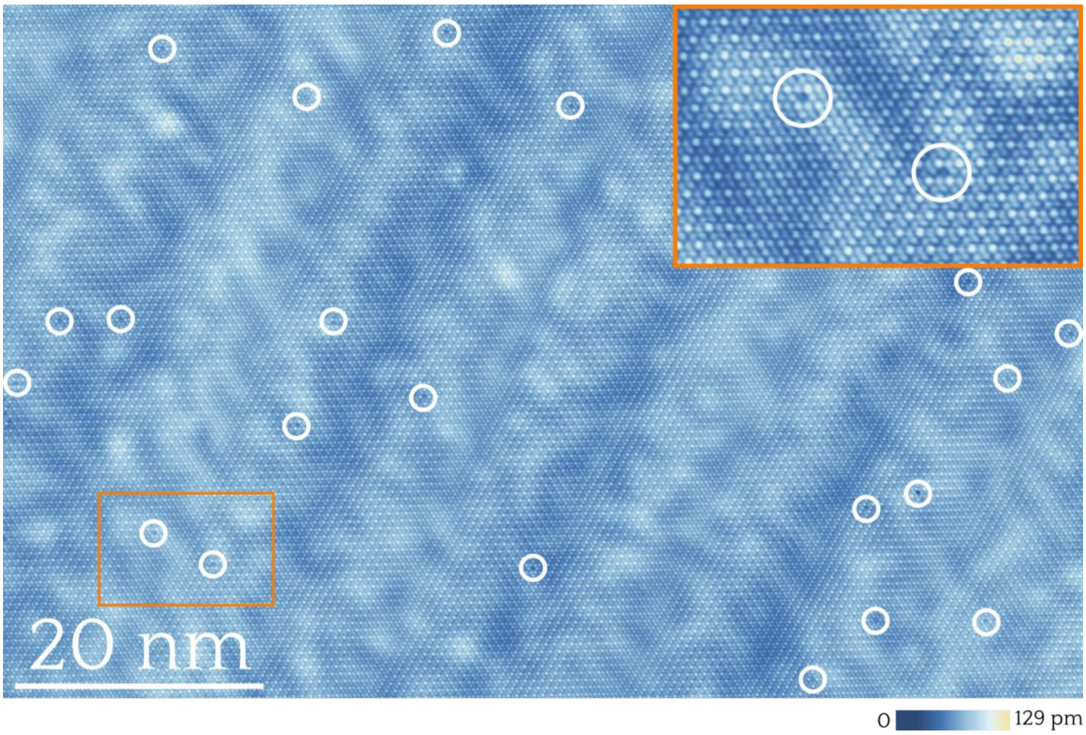

Fig. S3 | Defect density in single-layer $TiSe_2$/BLG. Typical atomically resolved STM image of our single-layer $TiSe_2$ films. White circles mark the position of point defects in the lattice. (V_s = -0.05 V, I_t = 60 pA). The inset shows a zoom in of the boxed region in the main image.

3. Large-scale electronic structure of single-layer $TiSe_2$

The electronic structure of single-layer $TiSe_2$ over a larger energy scale (± 2V) is shown in Fig. S4, compared to an *ab initio* calculation of the single-layer band structure reproduced from Ref. [S2]. The Y-axis (energy) between the two panels is rigidly shifted by 300 meV to account for the doping induced by the BLG substrate in the experiment. The dI/dV spectrum shows three main features, which derive from features in the band structure which are unrelated to the CDW state. A rather sharp onset in the occupied states labeled as V_3 corresponds to tunneling into the Se p_z orbitals, which in the monolayer occur at much lower energies than the bulk, as seen in the *ab initio* calculation. Features C_2 and C_3 in the unoccupied states correspond to Van Hove singularities in the t_{2g}-like bands of Ti *d* orbitals, which are also present in bulk $TiSe_2$ (Ref. [S3]). Note that the measured dI/dV features are weighted by tunneling matrix elements, which in this case are strongly affected by the in-plane *p*-

orbitals of the valence band pockets near the Fermi level. These orbitals have low tunneling probability to the tip, which requires a very sensitive measurement plotted in logarithmic scale to be appreciated (feature C_1 Fig 2(c) in the main text). The out-of-plane Se p_z orbitals at higher voltages give much larger contributions to tunneling, rendering the Fermi level features almost unobservable at the large bias voltage scales shown in Fig. S4.

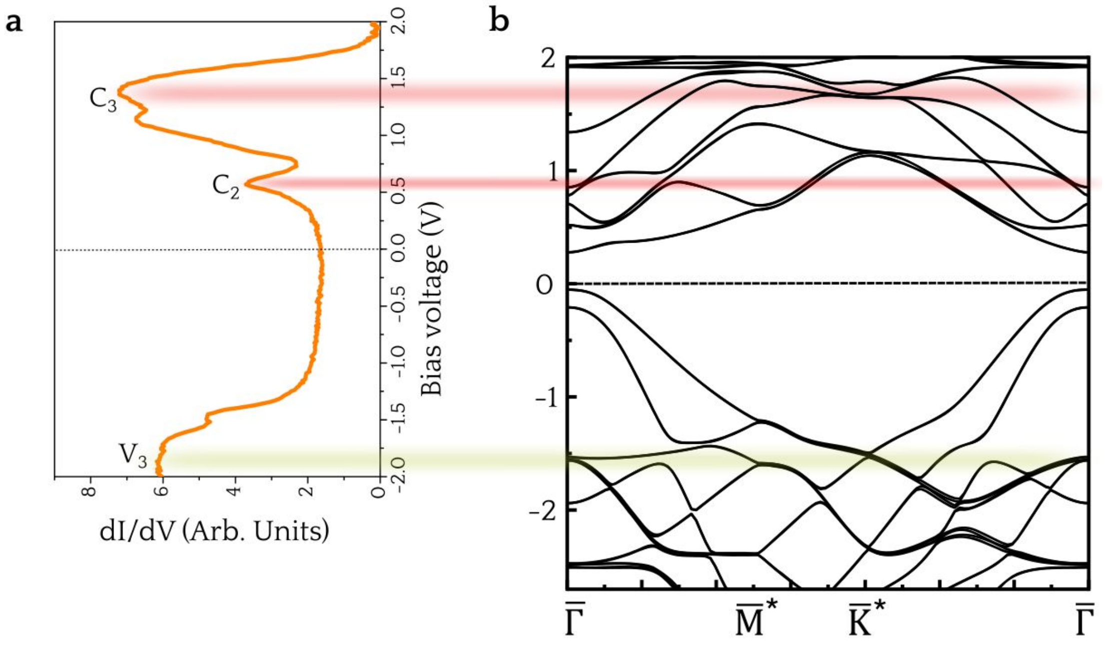

Fig. S4 | Large-scale tunneling spectroscopy. a, Representative large-scale dI/dV curve acquired on single-layer $TiSe_2$/BLG (T = 4.2 K). Further spectroscopic features labeled as V_3, C_2 and C_3 are shown, apart from those mentioned in the main manuscript (Fig. 2b) **b**, Calculated band structure for the monolayer $TiSe_2$ in the CDW state, adapted from Ref. [S2].

4. Primary and secondary order parameters of the CDW

The order parameters used in the main text are the primary order parameter Δ_n of M_1^- and three secondary order parameters ϕ^{SeUp}, ϕ^{Ti}, ϕ^{SeDo} of symmetry M_1^+. These order parameters are extracted as follows. The charge density in the CDW state can be decomposed into the original 1x1 lattice modulation and the period doubling 2x2 modulation of the CDW, $\rho(\bar{x}) = \rho_{1x1}(\bar{x}) + \rho_{2x2}(\bar{x})$. We can express the former in terms of the complex numbers $A_n^G(x)$ at the $\bar{G}_n$ Bragg peaks of the FFT,

$$\rho_{1x1}(\bar{x}) = 2\Re\sum_n A_n^G(\bar{x})e^{-i\bar{G}_n\bar{x}} = 2\sum_n \Re A_n^G(\bar{x})\cos(\bar{G}_n\bar{x}) + \Im A_n^G(\bar{x})\sin(\bar{G}_n\bar{x}).$$

The Lawler-Fujita method (see details below) enforces the phase of $A_n^G(x)$ to be constant. The 2x2 part of the charge modulation can be equivalently expressed in terms of the complex numbers $A_n^M(x)$ and $A_n^{M'}(x)$ at the $\bar{M}_n$ and $\bar{M}'_n = \bar{M}_n + \bar{G}_{n-1}$ peaks of the FFT,

$$\rho_{2x2}(\bar{x}) = 2\Re\Sigma_n\left[A_n^M(\bar{x})e^{-i\bar{M}_n\bar{x}} + A_n^{M'}(\bar{x})e^{-i\bar{M}'_n\bar{x}}\right]$$

$$= 2\sum_n \Re A_n^M(\bar{x})\cos(\bar{M}_n\bar{x}) + \Im A_n^M(\bar{x})sin(\bar{M}_n\bar{x}) + \Re A_n^{M'}(\bar{x})\cos(\bar{M}'_n\bar{x}) + \Im A_n^{M'}(\bar{x})sin(\bar{M}'_n\bar{x})$$

$$\equiv 2\sum_n \phi_n^{SeUp}(\bar{x})\, f_n^{SeUp}(\bar{x}) + \phi_n^{Ti}(\bar{x})f_n^{Ti}(\bar{x}) + \phi_n^{SeDo}(\bar{x})f_n^{SeDo}(\bar{x}) + \Delta_n(\bar{x})f_n^{\Delta}(\bar{x})$$

Here we have defined the secondary M_1^+ order parameters

$$\phi_n^{SeUp}(\bar{x}) = \Re A_n^{M'}(\bar{x}) - \frac{1}{2}\Re A_n^M(\bar{x}) + \frac{\sqrt{3}}{2}\Im A_n^M(\bar{x})$$

$$\phi_n^{Ti}(\bar{x}) = \Re A_n^{M'}(\bar{x}) + \Re A_n^M(\bar{x})$$

$$\phi_n^{SeDo}(\bar{x}) = \Re A_n^{M'}(\bar{x}) - \frac{1}{2}\Re A_n^M(\bar{x}) - \frac{\sqrt{3}}{2}\Im A_n^M(\bar{x})$$

with their corresponding site selective functions

$$f_n^{SeUp}(\bar{x}) = \tfrac{1}{3}\cos(\bar{M}'_n\bar{x}) - \tfrac{1}{3}\cos(\bar{M}_n\bar{x}) + \tfrac{1}{\sqrt{3}}sin(\bar{M}_n\bar{x}) \qquad (1)$$

$$f_n^{Ti}(\bar{x}) = \tfrac{1}{3}\cos(\bar{M}'_n\bar{x}) + \tfrac{2}{3}\cos(\bar{M}_n\bar{x}) \qquad (2)$$

$$f_n^{SeDo}(\bar{x}) = \tfrac{1}{3}\cos(\bar{M}'_n\bar{x}) - \tfrac{1}{3}\cos(\bar{M}_n\bar{x}) - \tfrac{1}{\sqrt{3}}sin(\bar{M}_n\bar{x}) \qquad (3)$$

These site selective functions, shown in Figs. S5 b-d, are all mirror *even*, and can therefore only represent a 2x2 modulation of the secondary phonon of M_1^+ symmetry (1-in-4 pattern in Fig. 1c). The imaginary part of $A_n^{M'}(x)$, on the other hand, is identified with the primary CDW order parameter with symmetry M_1^-,

$$\Delta_n(\bar{x}) = \Im A_n^{M'}(\bar{x})$$

$$f_n^{\Delta}(\bar{x}) = sin(\bar{M}'_n\bar{x}) \qquad (4)$$

since it represents mirror *odd* in-plane modulations, illustrated in Fig. S5a. Note that to determine without ambiguity the chirality of $\Delta_n(\bar{x})$ experimentally, all three sublattices (Se-up, Ti, Se-down) have to be resolved, as this sets the coordinate system and the reciprocal lattice vectors.

Since all three ϕ^{SeUp}, ϕ^{Ti}, ϕ^{SeDo} have the same symmetry, it is sufficient to consider the one of largest magnitude for the Ginzburg-Landau analysis, since the other two will be locked to the first by a quadratic coupling. Because of this, in the main text we take $\phi_n = \phi_n^{SeUp}$. Lattice translations a_n act on these order parameters with the operator $t_n = diag(e^{iG_1\bar{a}_n}, e^{iG_2\bar{a}_n}, e^{iG_3\bar{a}_n})$, which is explicitly $t_1 = diag(1,-1,-1)$, $t_2 = diag(-1,1,-1)$, $t_3 = diag(-1,-1,1)$. The different symmetry related ground states of the order parameters, shown in Fig. 1d, are explicitly defined as $\phi/\phi_0 = -\Delta/\Delta_0 = (1,1,1)$ for A, $\phi/\phi_0 = -\Delta/\Delta_0 = (1,-1,-1)$ for B, $\phi/\phi_0 = -\Delta/\Delta_0 = (-1,1,-1)$ for C and $\phi/\phi_0 = -\Delta/\Delta_0 = (-1,-1,1)$ for D. The primed states have the same ϕ but $\phi/\phi_0 = \Delta/\Delta_0$.

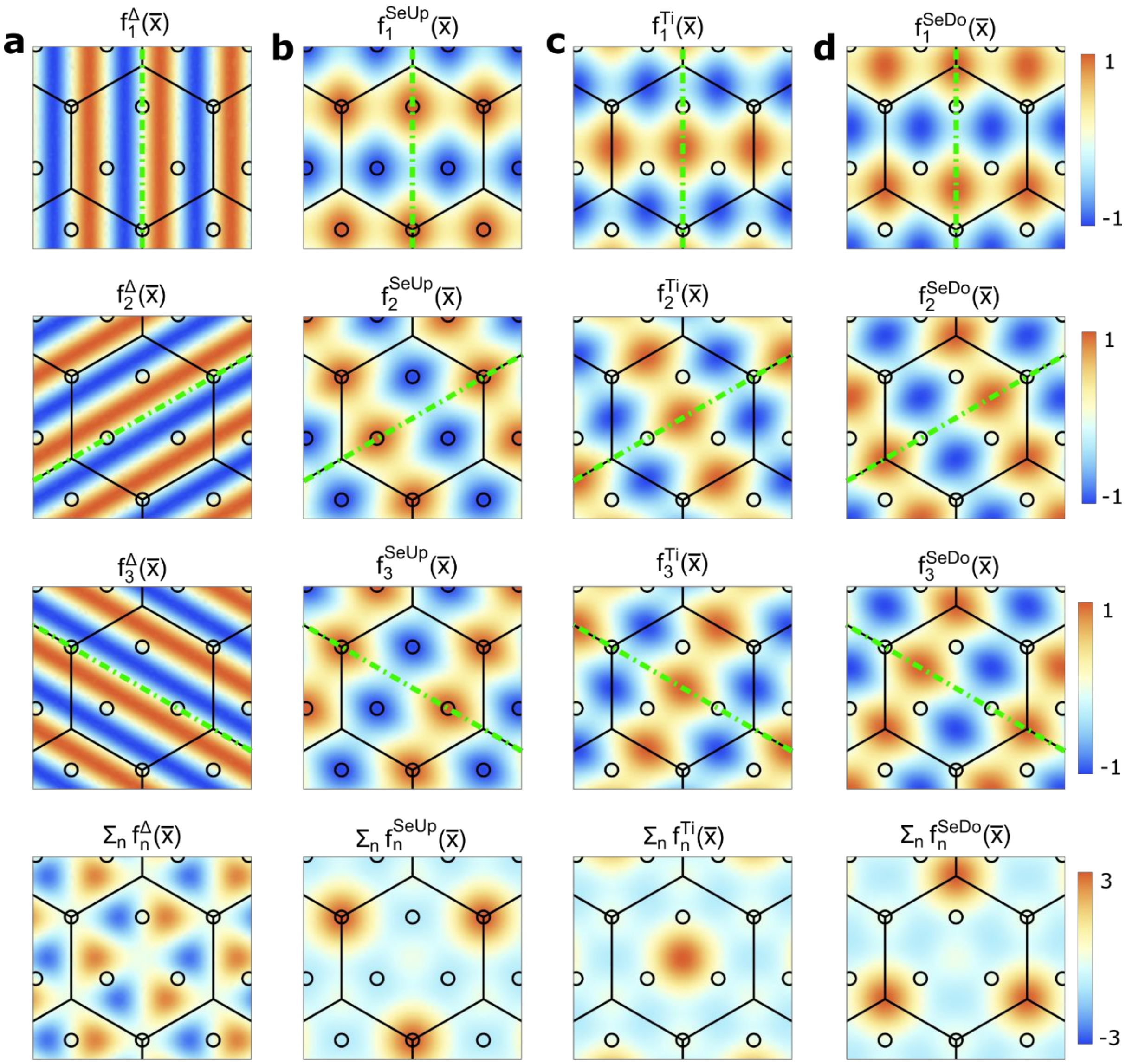

Fig. S5 | Primary and secondary 2x2 order parameters. a, Primary order parameter function components $f_n^{\Delta}(\bar{x})$ [eq. (4)]. **a**, Secondary order parameter site selective functions **b**, $f_n^{SeUp}(\bar{x})$ [eq. (1)] **c**, $f_n^{Ti}(\bar{x})$ [eq. (2)]and **d**, $f_n^{SeDo}(\bar{x})$ [eq. (3)]. The dotted lines represent the mirror σ_v parallel to the corresponding $\bar{M}_n$ vector.

To extract the primary and secondary order parameters, we need the spatially dependent complex amplitudes $A_n^M(x)$ and $A_n^{M'}(x)$ with well defined phases so that the real and imaginary parts can be separated. For this, we use geometric phase analysis (https://github.com/TAdeJong/pyGPA) and implement the LF algorithm [S4] to produce corrected STM images in perfect registry with the lattice, which enforces a constant phase of the complex numbers $A_n^G(x) \equiv A_n^G$. To fix the origin we note there are three points in the unit cell where $argA_n^G$ are approximately equal, which correspond to Ti, Se-Up and Se-Down atoms. We choose by convention that $-2\pi/3 < arg\ A_n^G < -\pi/3$ which selects the Ti site as origin and the order Se-Down-Ti-Se-Up as we move along (0,1) (see Fig. 1a). The $arg\ A_n^G(x)$

after the implementation of LF to the STM image of Fig.2a is shown in Fig.S6, which shows a constant value slightly bigger than $-2/3\pi$ for all three components. The corresponding 1x1 modulation is shown in Fig. S6b (see also Fig. 1d). The extracted 2x2 order parameters for this image are shown in Fig. 2c. Together with the extracted amplitude of the 1x1 lattice peaks ($|A_n^G| = 2.72$) one can build the total charge density $\rho(\bar{x}) = \rho_{1x1}(\bar{x}) + \rho_{2x2}(\bar{x})$, illustrated in Fig. 2d. The same procedure was used in Fig. 4.

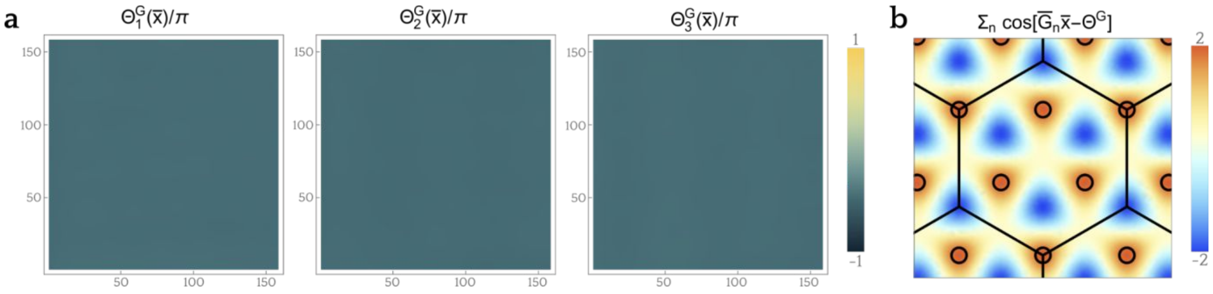

Fig. S6 | Geometric phase analysis and LF algorithm. a, Phase of the lattice Bragg peaks $\Theta_n^G(\bar{x}) = arg\ A_n^G(\bar{x})$ after implementation of the LF algorithm of an STM image from which Fig. 2a was extracted. It shows a constant value $\Theta_n^G(\bar{x}) = (-2/3 + 0.194)\pi$ throughout the field of view. **b**, Using the extracted phase Θ^G in a, the corresponding 1x1 lattice modulation $\rho_{1x1}(\bar{x})$ is reconstructed (see also normal state sketch in Fig. 1d).

5. STM imaging of the CDW pattern

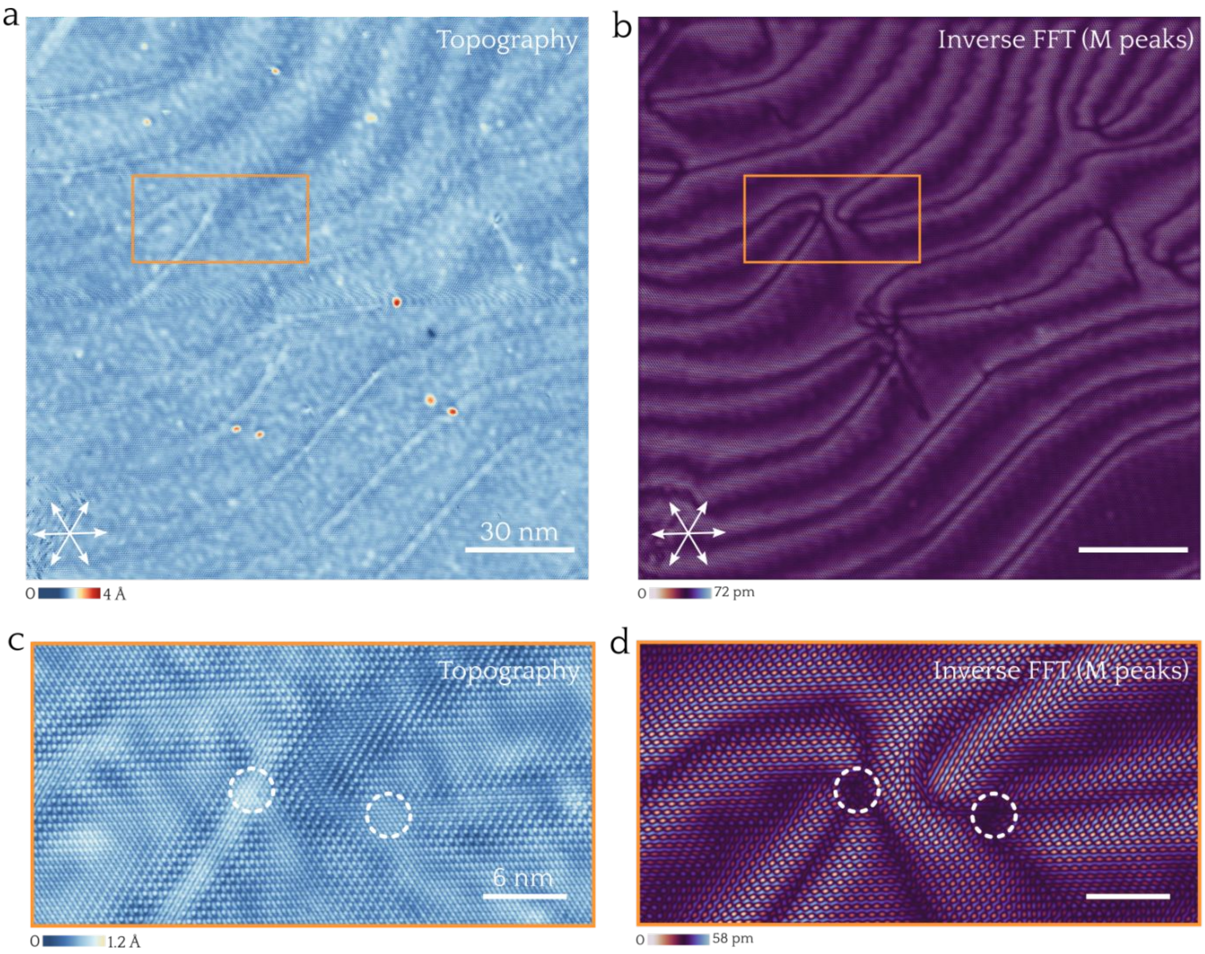

Fig. S7 | Complex 2D pattern in the CDW state of single-layer $TiSe_2$. **a** Atomically resolved STM topography of a 160 x 160 nm^2 region of single-layer $TiSe_2$ similar to tha shown in the main manuscript (V_s = -0.05 V, I_t = 50 pA). The white arrows indicate the crystal directions. **b** Inverse Fourier transform (FFT) of the CDW 2 x 2 peaks (M points) of the FFT (**c**) of the topography in **a**. **c** and **d** t shows zoomed-in images of the boxed region in the images in **a** and **b**, respectively.

6. Magnetic-field insensitivity of the CDW pattern

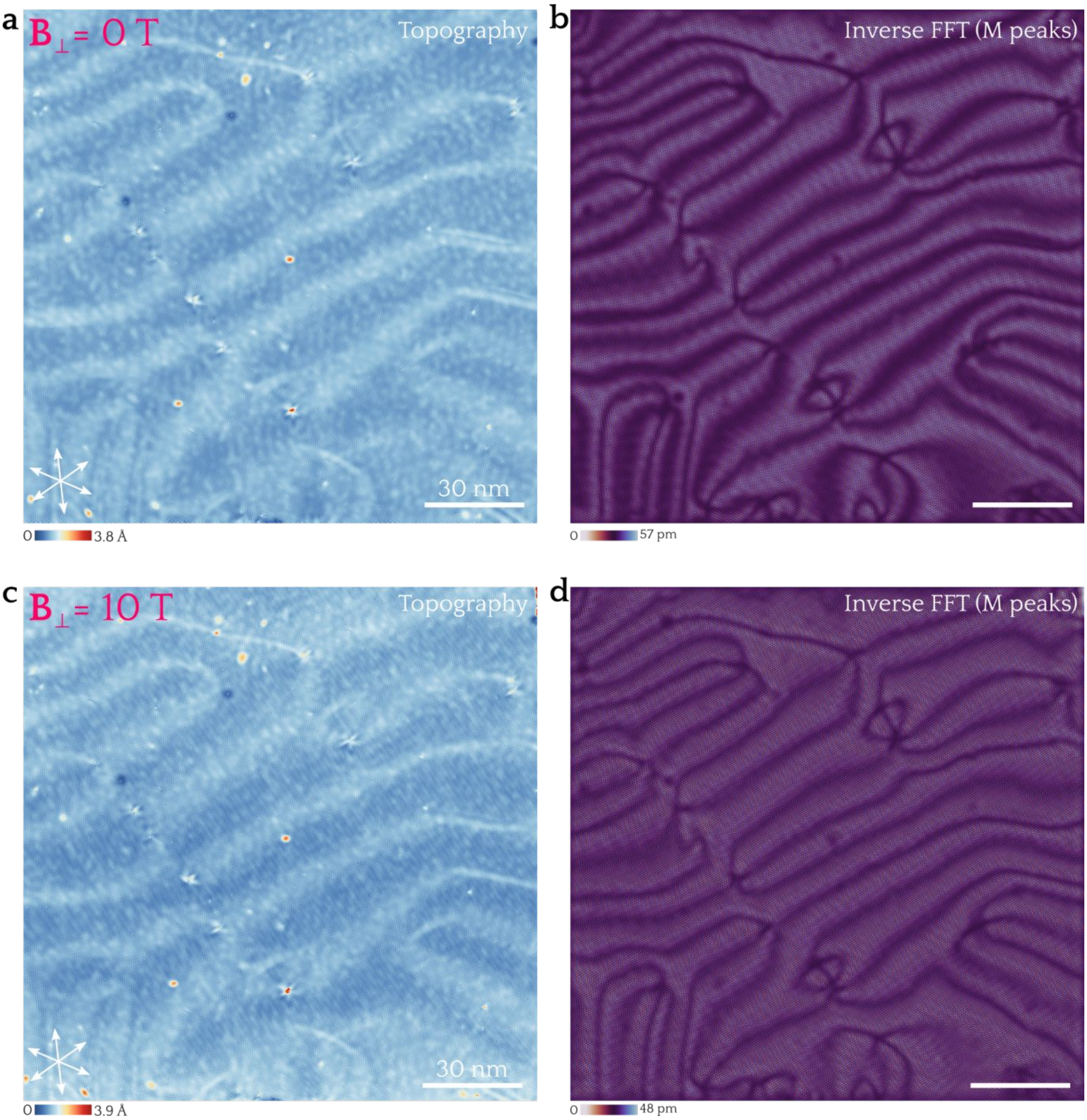

Fig. S8 | Magnetic-field dependent STM imaging. Atomically resolved STM topographs of single-layer $TiSe_2$ (**a**,**c**) and corresponding I-FFT of the CDW 2x2 peaks (**b**,**d**) acquired at different magnetic fields.

7. Temperature dependence of the CDW patterns

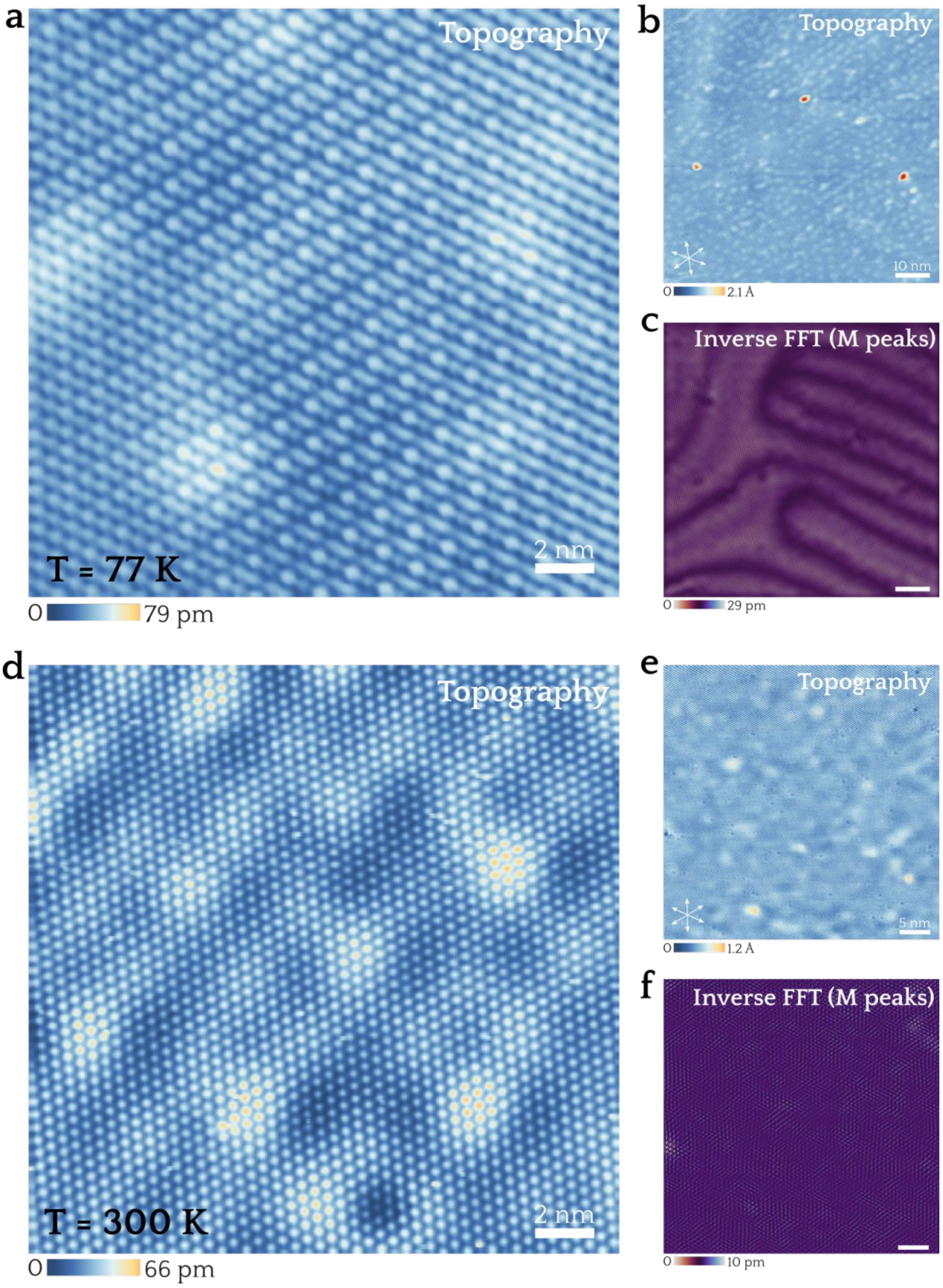

Fig. S9 | CDW pattern vs. temperature in monolayer $TiSe_2$. a,**b**, Short- and long-scale atomically resolved STM images at T = 77 K, respectively. Parameters: **a** (V_s = -0.2 V, I_t = 60 pA), **b** (V_s = -0.06 V, I_t = 100 pA). **c**, Corresponding I-FFT of the M points. **d**,**e**, Short- and long-scale atomically resolved STM images at T = 300 K, respectively. Parameters: **d** (V_s = 0.1 V, I_t = 600 pA) and **e** (V_s = -0.2 V, I_t = 1000 pA). **f**, Corresponding I-FFT of the M points.

8. Fourier-filtered STM imaging of relevant periodicities

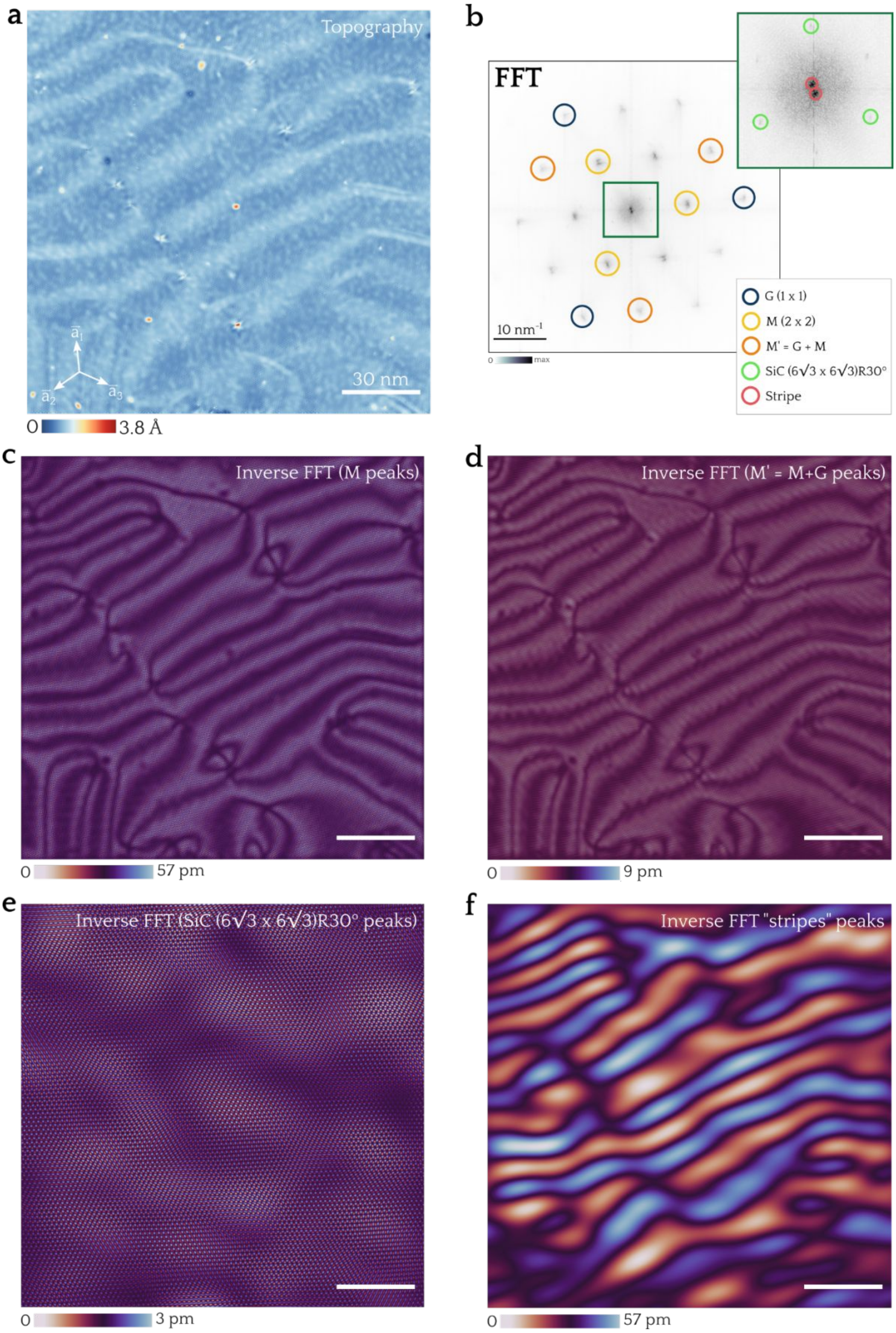

Fig. S10 | Contribution of other relevant points in *k*-space. a, Atomically resolved STM image shown in Fig. 2a. **b,** Corresponding FFT with showing the relevant peak features. I-FFT of the M (**c**), M' (**d**), SiC (**e**) and "stripe" (**f**) peaks.

References

S1 Horcas, I. *et al.*, Wsxm: A software for scanning probe microscopy and a tool for nanotechnology, *Rev. Sci. Instrum*. 78 (2007).

S2. Chen, P. *et al.* Charge density wave transition in single-layer titanium diselenide. *Nat. Commun.* **6**, 1–5 (2015).

S3. Benesh, G. A. *et al*. The pressure dependences of TiS_2 and $TiSe_2$ band structures. *J. Phys. C Solid State Phys.* **18**, 1595 (1985).

S4. Lawler, M.. *et al*, Intra-unit-cell electronic nematicity of the high-Tc copper-oxide pseudogap states, *Nature* **466**, 347 (2010).
